# TLR4-Mediated Immune Dysfunction Links MASLD and Parkinson’s Disease: Insights from an Omics-Based Network Analysis

**DOI:** 10.3390/neurosci7020028

**Published:** 2026-02-28

**Authors:** Christina Flourou, Nikolaos Dietis, Sotirios Tsiordas, Georgios Hadjigeorgiou, George D. Vavougios

**Affiliations:** 1 Medical School, University of Cyprus, Nicosia 2029, Cyprus; dietis.nikolas@ucy.ac.cy (N.D.); hadjigeorgiou.georgios@ucy.ac.cy (G.H.); dantevavougios@hotmail.com (G.D.V.); 2 4th Department of Internal Medicine, Medical School, University General Hospital Attikon, National and Kapodistrian University of Athens, 12462 Athens, Greece; sotirios.tsiodras@gmail.com

**Keywords:** MASLD, Parkinson’s disease, TLR4, SNPs, bioinformatics, statins, fenofibrate

## Abstract

**Background and aim**: Alterations in immune signaling have emerged as a key factor contributing to Parkinson’s disease pathophysiology. Increasing evidence also suggests that MASLD and Parkinson’s disease may share common immunological mechanisms. Among these, TLR4 has been linked to immune surveillance processes and inflammatory responses in both the central nervous system and the liver. The aim of our study was to delineate TLR4-mediated immune networks underpinning the molecular overlap between MASLD and Parkinson’s disease. **Methods**: Disease–disease and gene–disease associations were systematically retrieved from the DisGeNet database to map TLR4-related molecular networks across both conditions. Functional enrichment analyses were subsequently applied to identify biological pathways significantly associated with TLR4, including potential gene–drug interactions. Guided by these results, a scoping review of the literature was undertaken to summarize existing evidence addressing TLR4-dependent mechanisms in MASLD and Parkinson’s disease. **Results**: DisGeNet analysis indicated 978 shared genes and 39 SNPs shared between both diseases. TLRs, including TLR4-associated coreceptors such as CD14, are among these shared genes. Among these, TLR4 and its missense SNP rs4986791 emerged as key shared immunometabolic nodes linking both diseases. Among the shared SNPs identified in both diseases, we focused on TLR4, where the common variant was rs4986791. Gene set enrichment analysis revealed multiple biological processes associated with cytokine signaling, inflammation, and fibrogenesis. Gene–drug enrichment analysis identified statins and fibrates among the compounds enriched in TLR4-containing networks. **Conclusions**: These findings support a role for TLR4-associated pathways in linking immunometabolic processes across MASLD and Parkinson’s disease. Disruption of these pathways is associated with aberrant inflammatory regulation, with tissue-specific effects further contributing to the distinct molecular pathology observed in each condition. Consequently, modulation of TLR4 signaling represents a plausible strategy for the development or repositioning of disease-modifying interventions applicable to both conditions.

## 1. Introduction

With the rising prevalence of metabolic syndrome, metabolic dysfunction-associated steatotic liver disease (MASLD) has become the most common chronic liver disease in the Western world [1]. Several molecular changes support the “multiple hit” hypothesis in MASLD/MASH. These involve immune and inflammatory pathways that participate in the regulation of lipid metabolic processes [2]. Among these pathways, Toll-like receptor 4 (TLR4) has been shown to be a notable regulator of MASLD severity, studied through receptor activation by damage-associated molecular pattern (DAMP) proteins, such as eNAMPT. Extracellular NAMPT (eNAMPT), a damage-associated molecular pattern (DAMP) protein, activates the TLR4/NF-κB pathway, promoting multiorgan inflammation and fibrosis. This effect is especially pronounced in obesity, where elevated eNAMPT levels enhance TLR4 expression, leading to triglyceride accumulation, fatty acid oxidation, and progression from steatosis to fibrosis [3].

In part, these mechanisms may be related to exogenous immune challenge and lipid metabolism dysregulation; as such, TLR4 has been shown to be dysregulated in MASLD in conjunction with lipopolysaccharide challenge and palmitate administration. The presence of free fatty acids (FFAs), such as palmitate and stearic acid, and lipopolysaccharides (LPS) from Gram-negative bacteria induces NF-κB activation and TLR4 dysregulation. These signals are upregulated in obesity, contributing to hepatocellular inflammation and apoptosis. Elevated plasma LPS, FFAs, and TLR4 mRNA levels have been documented in MASH compared to MASLD. At the same time, inhibition of TLR4 by siRNA in hepatocytes could not suppress NF-κB activation, suggesting the participation of another TLR4-independent pathway [4].

TLR4 dysregulation may be a central event in MASLD pathogenesis from a genetic perspective, as its polymorphisms have been shown to contribute to disease severity. Genetic polymorphisms in TLR4, including Asp299Gly and Thr399Ile, contribute to hyporesponsiveness to endotoxins and are associated with metabolic diseases such as atherosclerosis, insulin resistance, and type 2 diabetes [5]. Notably, the Asp299Gly variant may offer relative protection from MASLD, especially in females [6].

Lipid metabolism and immune dysfunction have been increasingly implicated in the pathogenesis of neurodegenerative diseases. Several lipid metabolism–related genes, such as APOE-ε4 in Alzheimer’s disease (AD) and GBA in PD, influence cholesterol and ceramide pathways. In GBA mutation–associated PD, ceramide accumulation is correlated with α-synuclein aggregation and neuroinflammation. In GBA deficiency, lysosomal dysfunction leads to increased levels of extracellular vesicles (EVs), increased microglial uptake, propagation of toxic proteins, and neurodegeneration [7]. In parallel with lipid metabolism alteration, immune dysfunction caused by activation of the α-syn/TLRs/NF-κB/NLRP3 inflammasome axis results in dopaminergic neuronal damage. On the one hand, microglial clearance of misfolded α-synuclein protein is impaired; thus, this axis is activated. On the other hand, classical microglial activation (M1 phenotype) results in the release of pro-inflammatory cytokines and molecules that promote neuroinflammation in PD [8].

In PD, oxidative stress and immune dysregulation converge to promote α-synuclein aggregation. Misfolded α-syn impairs neuronal lipid transport, leading to lipid droplet accumulation and neuronal death. TLR4 expression is elevated both centrally and peripherally, contributing to neuroinflammation, especially in the context of gut dysbiosis and increased intestinal permeability [9].

TLR4 mediates gut–brain axis signalling. In dysbiosis, LPS-producing bacteria activate TLR4 via CD14 and MD2, promoting NF-κB-mediated cytokine release. These signals compromise the blood–brain ba, +rrier, enabling α-synuclein propagation from the gut to the CNS, where it contributes to Lewy body pathology and motor deficits [10,11,12]. Collectively, these findings suggest that TLR4 dysregulation contributes to the shared pathophysiological landscape of MASLD and PD, encompassing immune, lipid, and microbiome-driven mechanisms. A recent study by Asimakidou et al. (2025) [13] further supports this connection, reporting sex-specific associations between MASLD and PD risk. TLR4, an immune receptor independently associated with both diseases, represents an important focal point in immune processes that could be common to both diseases [13,14]. Notably, TLR4 may specifically account for the combination of immune and lipid dysregulation seen in both diseases, outlining the brain-liver axis [14]. Taken together, these findings suggest that TLR4 may act as a molecular nexus integrating metabolic, immune, and microbiome-driven signals across the liver and brain. To test this hypothesis, we performed a systematic omics-based exploration of TLR4 interactors and associated pathways.

## 2. Materials and Methods

**Figure 1:**
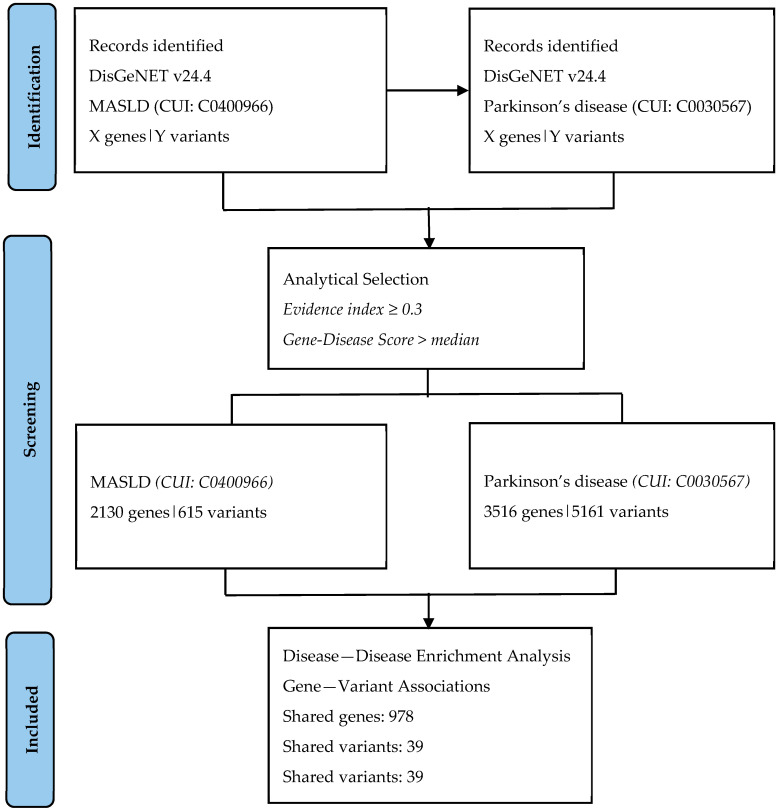
PRISMA-style flow diagram illustrating the identification and analytical selection of genes and variants associated with MASLD and Parkinson’s disease from DisGeNET (version 24.4). No filtering was applied at the DisGeNET interface level; selection criteria (evidence index ≥ 0.3 and gene–disease score above the median) were applied during downstream data processing.

Data acquisition: Data used in this study were retrieved from the DisGeNet database (version 24.4; Piñero et al., 2020) [15] to systematically identify genes and single nucleotide polymorphisms (SNPs) associated with MASLD. DisGeNet integrates manually curated and text-mined evidence from multiple sources to annotate gene–disease and variant–disease associations. Only associations with an evidence index ≥ 0.3 and a gene–disease score above the median value for each dataset were included to ensure data reliability. The search was conducted on 14 March 2025 and was subsequently re-checked in December 2025 for consistency; the term “non-alcoholic fatty liver disease (CUI: C0400966)” was used to represent MASLD due to terminological lag in the database. The data identification and selection workflow is summarized in a PRISMA-style flow diagram (Figure 1).

Disease–disease and SNPset enrichment analysis: The DisGeNet disease–disease enrichment tool was used to determine the overlap of genes and SNPs between MASLD and PD (CUI: C0030567). Shared genes were subsequently used to construct an interaction network analyzed for functional enrichment. SNP set enrichment was performed using the same shared variant list to identify associated biological processes. Gene set and gene–drug enrichment analyses were carried out via the Enrichr web server, applying Fisher’s exact test for overrepresentation and Benjamini–Hochberg false discovery rate (FDR) correction for multiple testing. Adjusted *p*-values < 0.05 were considered significant.

Protein–protein interaction (PPI) analysis: Shared genes were imported into the STRING database (version 12.0) for network generation [16]. Interactions were visualized using the confidence score cutoff of 0.7 (high confidence), integrating evidence from experimental, co-expression, and curated database sources to identify functionally enriched interaction clusters.

## 3. Results

### 3.1. Data Acquisition and Disease-Gene Associations

The DisGeNet database was accessed on 14 March 2025 for data collection and subsequently re-checked in December 2025 to confirm consistency. Disease enrichment analysis for non-alcoholic fatty liver disease (NAFLD), identified by the Concept Unique Identifier (CUI: C0400966), revealed a total of 2130 genes and 615 single nucleotide polymorphisms (SNPs) associated with the disease. Notably, due to terminological lag in DisGeNet, the outdated term “NAFLD” was used to represent the modern concept of MASLD. Similarly, disease–disease enrichment analysis for Parkinson’s disease (PD) (CUI: C0030567) yielded 3516 genes and 5161 SNPs that also showed associations with MASLD. A key finding was the identification of 978 overlapping genes and 39 shared SNPs between MASLD and PD, suggesting a significant genetic and molecular intersection between these two distinct disorders. Among these, a notable shared variant was the TLR4 missense polymorphism rs4986791, highlighting a potential common inflammatory or immunological mechanism.

### 3.2. Protein–Protein Interaction (PPI) Analysis

**Figure 2:**
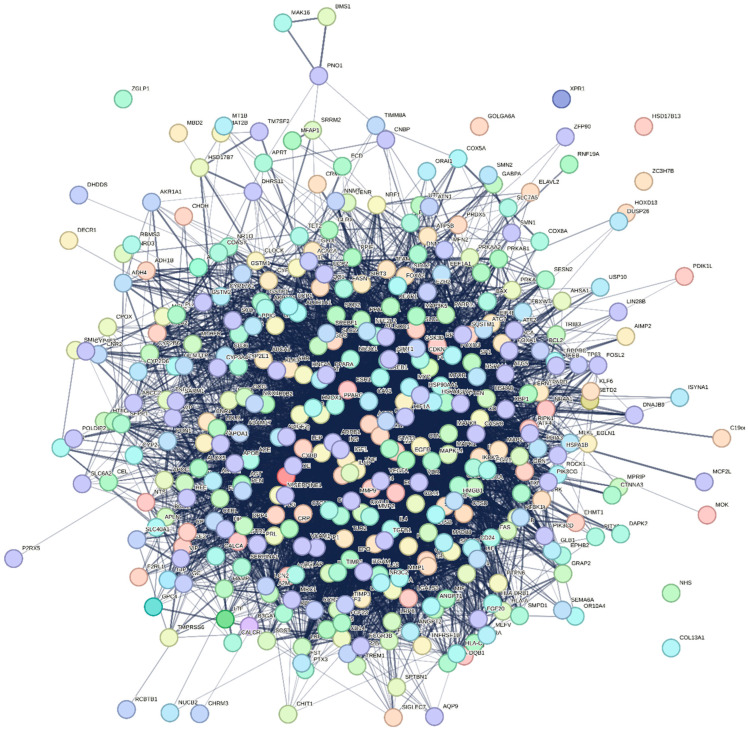
Protein–protein interaction (PPI) network of shared genes between MASLD and Parkinson’s disease. The TLR4-associated gene network was generated using the STRING database (v12). Nodes represent proteins and edges indicate predicted functional associations based on co-expression, experimental evidence, and curated database links. This visualization highlights the overall complexity and interconnectivity of the shared immunoinflammatory network, with TLR4 positioned within an inflammatory interaction neighborhood that includes key mediators such as IL6, TNF, and CD14.

The PPI network (Figure 2) revealed a dense interaction cluster centred on TLR4 and its co-receptors CD14 and MD2. Subsequent pathway enrichment highlighted these genes within cytokine- and NF-κB-mediated signalling processes, emphasizing TLR4’s integrative role in immunometabolic regulation. This suggests that the shared gene set is not a random overlap but may represent biologically meaningful functional modules involved in both MASLD and PD. Table 1 summarizes the characteristics of the shared gene TLR4 and the variant rs4986791 between MASLD and PD. The Disease Specificity Index (DSI) for TLR4 was 0.45, indicating that this gene is moderately specific in terms of disease associations, while the Disease Pleiotropy Index (DPI) was 0.03, suggesting low pleiotropy. This indicates that TLR4 is primarily associated with a narrow spectrum of diseases. The SNP rs4986791 is located on chromosome 9 and is classified as a missense variant, which typically alters protein function and may contribute to the pathogenic processes of MASLD and PD.

Due to database export limitations, only the retrievable records from the searches were downloaded and are provided in the Supplementary Materials. The NAFLD-associated genes and variants (search ID: C0400966) are presented in Files S1 and S2, respectively. The Parkinson’s disease-associated genes and variants (search ID: C0030567) are presented in Files S3 and S4. Disease enrichment analysis results are provided in Table S1.

### 3.3. Pathway Enrichment Analysis

Gene set enrichment analysis was performed using the Enrichr web server, focusing on TLR4-associated pathways. The analysis revealed ten significantly enriched biological processes, all of which were heavily implicated in the regulation and propagation of inflammation, as shown in Table 2. These include the regulation of inflammatory response, positive regulation of cytokine production, and cytokine-mediated signalling pathways, among others. Prominent immune and inflammatory mediators, such as IL1, IL6, TNF-α, and TLR7, are involved in these pathways, highlighting the central role of TLR4 in modulating immune responses.

As shown in Table 2, some of the most significantly enriched Gene Ontology (GO) terms include Regulation of inflammatory response (GO:0050727), Cytokine-mediated signalling pathway (GO:0019221), and Inflammatory response (GO:0006954), all with adjusted p-values below 2.4 × 10^−26^. These pathways highlight TLR4’s critical role as a sensor and mediator in the innate immune system, where it modulates downstream cytokine production and inflammatory cascades. Additionally, pathways such as Positive regulation of cytokine production (GO:0001819) and Positive regulation of transcription by RNA polymerase II (GO:0045944) suggest that TLR4 activation not only triggers inflammation but also enhances gene expression programs that sustain and amplify inflammatory signalling. The involvement of TLR4 in Positive regulation of macromolecule metabolic processes and Gene expression links inflammation to broader metabolic dysregulation, a key feature of MASLD. The high significance levels (adjusted *p*-values ranging from 1.6 × 10^−32^ to 9.1 × 10^−24^) collectively indicate TLR4 as a central hub in chronic inflammation. This suggests that the shared genetic architecture between MASLD and PD may be driven, at least in part, by common inflammatory mechanisms. The activation of TLR4-related pathways could contribute to both hepatic inflammation in MASLD and neuroinflammatory responses in PD. Thus, the enrichment results not only validate the relevance of TLR4 in the context of systemic inflammation but also provide mechanistic insight into the possible molecular crosstalk between metabolic and neurodegenerative disorders.

To evaluate the sensitivity of STRING-derived networks to interaction confidence thresholds, we examined the same network using minimum required interaction scores of 0.4, 0.7, and 0.9. Increasing stringency led to a gradual reduction in the number of edges (47, 39, and 32 edges, respectively), while the number of nodes remained constant (11 nodes), as shown in Table 3. Despite this reduction, the networks remained highly connected, with average node degrees of 8.55, 7.09, and 5.82, and high average local clustering coefficients (0.918, 0.845, and 0.836), respectively. Importantly, all networks showed strong protein–protein interaction enrichment compared to random expectation (PPI enrichment *p*-values ranging from 2.38 × 10^−13^ to 5.28 × 10^−8^), indicating that the observed connectivity is biologically meaningful and robust across confidence thresholds.

**Figure 3:**
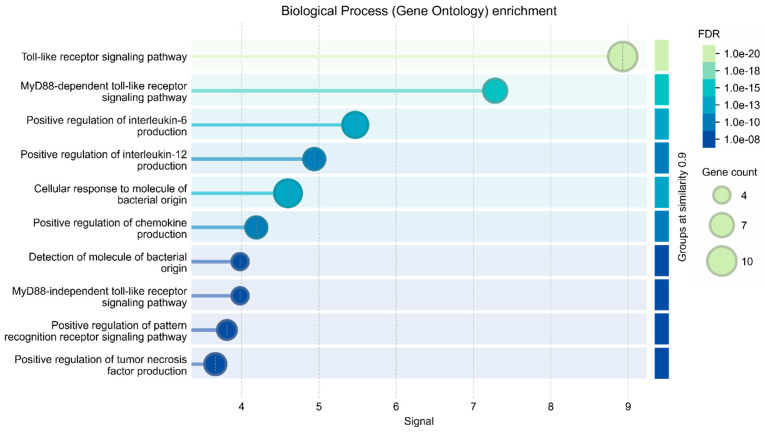
Degree and centrality distribution at a STRING confidence cutoff of 0.9. Histogram showing the distribution of node connectivity under a stringent STRING interaction confidence threshold (0.9), illustrating a right-skewed network topology characterized by a small number of highly connected hub nodes and a larger proportion of low-degree nodes.

Analysis of degree and centrality distributions at the highest confidence threshold (0.9) revealed a right-skewed topology, characterized by a small number of highly connected hub nodes and a larger proportion of low-degree nodes (Figure 3), indicating that hub dominance persists even under stringent interaction filtering.

### 3.4. Gene–Drug Enrichment Analysis

Building upon the initial pathway enrichment analysis, a gene–drug enrichment analysis was performed using the Enrichr web server to identify potential therapeutic compounds targeting the TLR4-associated gene network. This analysis specifically focused on drugs known to interact with the genes implicated in MASLD. Among the top-ranking compounds, statins emerged as the leading candidates, suggesting their potential as first-line therapeutic agents for MASLD because of their anti-inflammatory and lipid-lowering properties. Additionally, fibrates, particularly bezafibrate and fenofibrate, were significantly enriched in the TLR4-associated network, further supporting their relevance in MASLD treatment (Table 4). These findings highlight the therapeutic potential of modulating TLR4-related pathways, particularly in metabolic and inflammatory contexts.

## 4. Discussion

In this study, we identified overlapping gene expression patterns, enriched pathways, and polymorphisms between MASLD and PD. Our integrative omics approach identified a shared TLR4 variant (rs4986791) and multiple immune/metabolic pathways that intersect in MASLD and PD. These include cytokine signalling, lipid metabolism, and oxidative stress. The gene–drug enrichment data support a potential therapeutic role for statins and fibrates, which target these shared networks.

Toll-like receptors (TLRs) are pattern recognition receptors (PRRs) expressed on immune and parenchymal cells, including hepatocytes, microglia, and neurons. Among them, TLR2 and TLR4 are especially potent in recognizing bacterial components, such as lipoproteins and LPS, respectively [17,18,19,20,21]. Upon activation by PAMPs or DAMPs, TLR4 initiates a signalling cascade through NF-κB, promoting inflammation, autophagy, and apoptosis [22]. Besides exogenous ligands such as PAMPs, endogenous alarmins like HMGB1 can also activate this pathway, sustaining inflammatory responses in MASLD/MASH.

The rs4986791 polymorphism in TLR4 leads to a missense variant that alters receptor function, reducing host responsiveness to endotoxins and impairing intestinal immune surveillance. In both MASLD and PD, such dysregulation could promote chronic inflammation via aberrant TLR4/NF-κB signalling and disruption of the gut–liver–brain axis [23,24,25,26,27], with tissue-specific effects depending on cellular context. First, healthy individuals express low levels of Toll-like receptor 4 on their cell surfaces. TLR4 activity depends not only on the quantity of receptors but also on their functionality. This variant-induced dysfunction activates downstream TNF-α signaling and amplifies microbial dysbiosis–driven inflammation. Specifically, mediating the TLR4/TBK1/NF-κB/TNF-α signalling pathway, inflammation induces peripheral immune activation, and inflammatory factors cross the blood–brain barrier causing neuroinflammation and lower levels of dopamine (DA) and 5-hydroxytryptamine (5-HT) [27]. This pathway is well studied in the pathophysiology of PD.

In MASLD/MASH, liver steatosis leads to inflammation and progressive fibrosis through lipotoxicity. Lipotoxicity provokes hepatocyte death; therefore, death-associated molecular patterns (DAMPs) are released from damaged cells. Afterward, the secretion of TGF-β and tissue inhibitor of metalloproteinase leads to liver fibrosis and disease progression [28].

In both MASLD and PD, the microbiota–gut–brain axis is strongly implicated in disease pathophysiology. Microbial dysbiosis, the expression of LPS molecules on Gram-negative bacteria, and bacterial translocation activate TLR4. First, LPS molecules expressed by Gram-negative bacteria are recognized by CD14 and transferred to the MD2–TLR4 complex, thereby activating the TLR4 pathway [29].

The immune aspect of idiopathic PD has been increasingly recognized in the literature [30]. Microglial TLRs are key players in neurodegeneration, as they mediate inflammation-induced microgliosis and neuroinflammation. TLRs are upregulated in the brains of patients with Alzheimer’s disease, amyotrophic lateral sclerosis, and PD. In animal and human microglial cells surrounding senile plaques, there is an increased expression of TLR2, TLR4, and the TLR co-receptor CD14 [31].

The TLR4/NLRP3/caspase-1 axis is increasingly recognized as a critical pathway in PD. Activation of TLR4 by α-synuclein aggregates promotes inflammasome formation, mitochondrial dysfunction, and dopaminergic neuronal loss [32,33,34,35,36,37]. This aligns with our findings of TLR4 pathway enrichment and supports a central role for innate immune dysfunction in PD.

Our findings point towards a simvastatin-induced network that contains genes involved in immunological processes associated with both diseases, including TLR4. Simvastatin is used for the treatment of MASLD according to EASL and AASLD Clinical Practice Guidelines [38,39]. Besides their lipid-lowering effect, statins also have cardiovascular protective effects through their anti-inflammatory, antioxidative, and anti-thrombotic properties, affecting portal hypertension, cirrhosis progression, and hepatocellular carcinoma [40,41,42,43,44,45,46,47]. Notably, the liver may be an important site for alpha-synuclein clearance from the brain and peripheral sources; drugs such as statins with relevance to both diseases may also preserve this underexplored homeostatic brain-liver axis [48,49].

The precise role of statins in idiopathic PD remains controversial. Statins affect the neuropathology of PD in both directions, harmful or protective. On the one hand, statins have a neuroprotective role against the development and progression of PD. In experimental models, simvastatin can cross the blood–brain barrier (BBB) and inhibit the activation of NF-κB pro-inflammatory cytokines TNF-α, IL-1β, and IL-6 in stimulated rat astrocytes, suppressing small G proteins and other pro-inflammatory molecules in glial cells [50]. Simultaneously, oxidized metabolites of cholesterol accelerate α-syn aggregation, leading to dopaminergic loss and motor deficits. Therefore, by using lipid-lowering drugs such as statins, reduced cholesterol levels could decrease α-syn aggregation and slow the progression of PD [51].

Statins have multiple pleiotropic effects, one of which is their immunomodulatory efficacy through regulatory T cell (Treg) enhancement; they eliminate immune responses to autoantigens, preventing PD [52]. In addition, statins inhibit ERK1/2 MAP kinase and treat L-DOPA-induced dyskinesia, an adverse event that is observed during chronic dopamine replacement therapy in PD [53,54]. Oral administration of simvastatin and pravastatin inhibits p21ras activation, exerting a protective effect on the substantia nigra and glial cells, reducing dopaminergic neuron damage and improving motor defects in MPTP-intoxicated mice [55]. Despite the beneficial effects of statins described in some studies, data from experimental models are contradictory. Higher levels of cholesterol have been shown to be associated with a decreased risk of PD, with a dose–effect relationship, in a prospective population-based cohort study [56]. Cholesterol is a major component of neuronal cell membranes and synapses and is essential for neuronal structure and function; however, it is not known whether neurons in PD differ in cholesterol levels compared with neurons from healthy individuals. Additionally, carriers of the APOE ε2 allele, which is associated with lower plasma levels of total cholesterol, had an increased risk of developing PD, and statins had no impact on disease severity assessment [57,58,59].

Statins may negatively affect Parkinson’s disease (PD) by causing CoQ10 isoenzyme deficiency, linked to CoQ2 gene polymorphisms—which disrupt mitochondrial function and increase apoptotic proteins [60]. In simvastatin-treated GT1-7 hypothalamic cells, cholesterol depletion led to reduced SREBP2 production, MAPK pathway inhibition, and elevated caspase levels, similar to the diabetic brain’s insulin-resistant state and impaired autophagy [61]. These changes promote early and late apoptosis, enhancing cell death and PD pathology, further supported by the harmful effects of statin-induced mevalonate metabolites [62].

In our Gene–Drug Enrichment analysis, fibrates, particularly bezafibrate and fenofibrate, were significantly enriched in the TLR4-associated networks. The effectiveness of fibrates, especially fenofibrate and bezafibrate, in MASLD is well established through PPARα expression in the liver and modulation of fat metabolism. The multifaceted role of fibrates in MASLD as antioxidant, anti-inflammatory, and antifibrotic factors regulates disease progression [63]. Fenofibrate-related PPARα activation affects the expression of genes encoding modulator factors that promote oxidative stress and inflammation. For instance, TNF-α, monocyte chemoattractant protein-1, and vascular cell adhesion molecule-1 production are inhibited, resulting in improvement in steatosis grade and inflammatory pathogenesis. Furthermore, fibrates limit the levels of interleukin-1 (IL-1) and C-reactive protein expression. These drugs also exert effects on the NF-κB pathway, reducing stellate cell and macrophage liver infiltration [63,64].

First, fenofibrate stimulates lipoprotein lipase activity, reduces very low-density lipoprotein (VLDL) production, and inhibits insulin resistance and hyperinsulinemia [65,66]. As a result, decreased circulating levels of fatty acids, triglycerides, and diacylglycerol accumulation are observed. Adiponectin levels are lower in diabetes mellitus and metabolic syndrome. Adiponectin decreases aldehyde oxidase 1 enzyme and connective tissue growth factor in vitro [67]. Therefore, in MetS, lower levels of adiponectin promote fibrinogenesis and cell damage. Fibrates play an important role in this process by increasing plasma levels of adipokines and preventing liver fibrosis [68].

Despite the role of fibrates in steatosis and fibrosis regression, their role in hepatic microcirculation has been observed in mouse models. In mice with MASLD induced by a high-fat diet, treatment with fibrates tends to improve tissue oxygenation and, especially, the microvascular patency of hepatic sinusoids, in which steatosis causes narrowing. Sinusoidal microcirculation and vascular tone are improved by promoting nitric oxide production and suppressing endothelin-1 (ET-1) [69].

In contrast, fenofibrate and bezafibrate appear to have beneficial effects in ameliorating PD symptoms and MASLD. Their roles as activators of peroxisome proliferator receptor alpha (PPAR-α) may account for some of these neuroprotective effects. Notably, experimental models of rats receiving fenofibrate orally and rotenone intraperitoneally showed improvements in memory loss and depressive behavior. Similarly, the administration of fibrates, especially fenofibrate, attenuated the aggregation of α-synuclein in the striatum in rotenone-induced parkinsonian rats [70]. Similar studies in experimental parkinsonian rats induced by 1-methyl-4-phenyl-1,2,3,6-tetrahydropyridine (MPTP) and 6-hydroxydopamine (6-OHDA), treated with fenofibrate or bezafibrate, revealed improvement of motor activity using actimetry [71].

PPAR-α agonists can also regulate cell proliferation and differentiation, as well as apoptosis [72]. In MPTP-lesioned models—which exhibit depletion of glutathione (GSH), superoxide dismutase (SOD), and lipid peroxidation (LPO) in the brain—these changes are correlated with increased specific activity of lipid peroxidation in both the striatum and midbrain, suggesting an association between learning and memory deficits and oxidative stress. The PPAR-α agonist fenofibrate decreases oxidative stress by normalizing GSH and SOD activity and mitigating LPO [72]. Hence, PPAR-α activation may attenuate inflammatory signaling and oxidative stress, supporting its potential neuroprotective role in PD-related neuroinflammation.

Overall, these mechanistic links underscore the value of integrative approaches for defining cross-domain biological signatures that connect systemic metabolic dysfunction with brain pathology. More broadly, our systems-biology framework aligns with an emerging translational neuroscience paradigm that leverages multi-modal datasets to define robust biological “signatures” linking systemic or environmental influences to neural and cognitive outcomes. Recent work has demonstrated how complex external exposures (e.g., peer-environment context) can map onto distinct neural, cognitive, and psychopathological signatures, underscoring the value of signature-based approaches in modern neuroscience [73]. In parallel, our study maps immunometabolic features of MASLD onto neuroinflammatory/neurodegenerative risk through liver–brain axis–relevant network signatures, supporting the identification of cross-domain biomarkers that may inform risk stratification and prevention strategies in PD.

Our gene–SNP analysis revealed that TLR4 and the specific nucleotide polymorphism of TLR4, SNP rs4986791, are correlated with the mechanism of action of fibrates. This result underlines that this SNP, which causes dysfunction of TLR4 and activation of inflammatory pathways and oxidative stress, is a significant part of the MASLD and PD pathophysiological networks. Furthermore, gene–drug analysis showed that fibrates, by interfering with TLR4 network pathways, are associated with MASLD and PD prevention, progression, and treatment. Given TLR4’s central role as an immunometabolic integrator, circulating markers of TLR4 activation (such as CD14, LBP, or soluble TLR4) could serve as measurable proxies of liver–brain axis inflammation in clinical cohorts of MASLD or PD. Establishing such biomarkers could facilitate patient stratification, monitoring of treatment response, and early identification of individuals at risk for neuroinflammatory complications. Importantly, as the clinical definition of MASLD continues to evolve by incorporating cardiometabolic criteria (e.g., obesity, type 2 diabetes, hypertension, dyslipidemia, and insulin resistance), future omics studies may need to refine MASLD-associated gene sets to better capture metabolic nuances that may not be fully represented by legacy NAFLD-based annotations. Taken together, our integrative analyses highlight TLR4-associated immunometabolic pathways as a plausible liver–brain axis link between MASLD and PD, motivating future validation studies and biomarker-guided therapeutic strategies.

## 5. Conclusions

Our findings demonstrate that multiple immune and metabolic pathways converge in MASLD and Parkinson’s disease, underscoring a shared immunometabolic basis between hepatic and neurodegenerative pathology. Through a data-driven omics approach, we identified TLR4 and its missense variant rs4986791 as key molecular nodes bridging the two conditions. To our knowledge, this is the first integrative omics study to implicate TLR4 in both MASLD and PD, revealing a common immunometabolic signature. The contribution of TLR4 to these shared pathways is multifaceted and context-dependent, reflecting tissue-specific homeostatic roles rather than a simple activation–inhibition dichotomy. Our network analysis strongly supports an association between elevated or dysfunctional TLR4 expression and systemic dysregulation, reflected in hepatic inflammation in MASLD and neuroinflammation in PD; nonetheless, longitudinal and experimental validation is required to determine whether TLR4 dysfunction causally drives progression from hepatic inflammation to neurodegeneration. Collectively, these insights highlight TLR4 signaling as a potential mechanistic link and therapeutic target within the liver–brain axis, offering a foundation for future translational and clinical investigations.

## Figures and Tables

**Table 1:** Shared gene and variant in MASLD and PD. Disease Specificity Index (DSI) ranges from 0.25 to 1. The DSI parameter reflects the number of diseases associated with a gene or variant. For instance, a DSI of 1 indicates an association with only one disease. Disease Pleiotropy index (DPI) ranges from 0 to 1. The value of this parameter explains whether the multiple diseases associated with the gene or variant are similar.

Index Disease	Associated Disease	Gene	Variant	DSI	DPI	Consequence	Chromosome
Non-alcoholic fatty liver disease	PD	TLR4	rs4986791	0.45	0.03	missense variant	9

**Table 2:** Enriched biological processes associated with TLR4-containing networks shared between MASLD and Parkinson’s disease. Top ten Gene Ontology (GO) biological processes identified through Enrichr pathway enrichment analysis of TLR4-associated genes. The table lists GO term names, nominal and adjusted *p*-values, indicating significance after multiple-testing correction. Adjusted *p*-values were calculated using the Benjamini–Hochberg false discovery rate (FDR) correction. These processes converge on immune activation, cytokine production, and regulation of inflammatory signaling.

Index	Pathway Association	*p*-Value	Adjusted *p*-Value
1	Positive Regulation Of Multicellular Organismal Process (GO:0051240)	4.018 × 10^−36^	1.616 × 10^−32^
2	Regulation Of Inflammatory Response (GO:0050727)	5.454 × 10^−34^	1.097 × 10^−30^
3	Positive Regulation Of DNA-templated Transcription (GO:0045893)	4.856 × 10^−32^	6.510 × 10^−29^
4	Positive Regulation Of Macromolecule Metabolic Process (GO:0010604)	1.467 × 10^−30^	1.475 × 10^−27^
5	Cytokine-Mediated Signaling Pathway (GO:0019221)	2.485 × 10^−29^	1.999 × 10^−26^
6	Inflammatory Response (GO:0006954)	3.460 × 10^−29^	2.319 × 10^−26^
7	Positive Regulation Of Cytokine Production (GO:0001819)	1.616 × 10^−28^	9.283 × 10^−26^
8	Positive Regulation Of Transcription By RNA Polymerase II (GO:0045944)	1.333 × 10^−26^	6.203 × 10^−24^
9	Regulation Of Cold-Induced Thermogenesis (GO:0120161)	1.388 × 10^−26^	6.203 × 10^−24^
10	Positive Regulation Of Gene Expression (GO:0010628)	2.260 × 10^−26^	9.091 × 10^−24^

**Table 3:** STRING network metrics at different confidence thresholds. Increasing stringency led to a gradual reduction in the number of edges (47, 39, and 32 edges, respectively), while the number of nodes remained constant (11 nodes).

Cutoff	Nodes	Edges	Avg Degree	Avg Clustering	PPI-*p* Value
0.4	11	47	8.55	0.918	2.38 × 10^−13^
0.7	11	39	7.09	0.845	2.35 × 10^−11^
0.9	11	32	5.82	0.836	5.28 × 10^−8^

**Table 4:** **Gene–drug enrichment analysis for compounds targeting the TLR4-associated network.** Top drugs identified by Enrichr analysis as significantly enriched within the TLR4-related gene set. The *p*-values and adjusted *p*-values indicate statistical significance of enrichment, while the odds ratio and combined score reflect the strength and consistency of each association. The analysis revealed that Bezafibrate and fenofibrate emerged as the leading fibrates interacting with the TLR4-inflammatory network.

Drug	*p*-Value	Adjusted *p*-Value	Odds Ratio	Combined Score
bezafibrate CTD 00005506	2.462 × 10^−60^	5.762 × 10^−58^	43.49	5969.59
fenofibrate CTD 00006620	4.749 × 10^−58^	9.945 × 10^−56^	42.05	5550.16

## Data Availability

The data presented in this study are available in the article and Supplementary Materials.

## Supplementary Materials

The following supporting information can be downloaded at: https://www.mdpi.com/article/10.3390/neurosci7020028/s1. File S1: NAFLD genes search_C0400966, File S2: NAFLD variants search_C0400966, File S3: Parkinson’s genes search_C0030567, File S4: Parkinson variants search_C0030567, Table S1: Disease Disease enrichment PD and NAFLD.

## Abbreviations

The following abbreviations are used in this manuscript:

PD	Parkinson’s Disease
MASLD	Metabolic dysfunction-associated steatotic liver disease
